# How does environmental punishment affect regional green technology innovation?—Evidence from Chinese Provinces

**DOI:** 10.1371/journal.pone.0288080

**Published:** 2023-07-21

**Authors:** Wei Wang, Jie Wen, Zhigao Luo, Wenyi Luo

**Affiliations:** 1 School of Finance, Chongqing Technology and Business University, Chongqing, China; 2 Research Center for Economy of Upper Reaches of the Yangtze River, Chongqing Technology and Business University, Chongqing, China; 3 School of Business Administration, Chongqing Technology and Business University, Chongqing, China; University of Naples Federico II: Universita degli Studi di Napoli Federico II, ITALY

## Abstract

As an important means of environmental regulation, environmental punishment lacks in empirical evidence on its impact on regional green technology innovation in China. Based on panel data of 30 provinces in China from 2010 to 2020, this paper systematically examines the relationship between environmental punishment and regional green technology innovation. It is found that environmental punishment has the quantity and quality enhancing effects on regional green technology innovation, and the quantity enhancing effect is greater than the quality enhancing effect. There is no significant effect difference between monetary punishment and non monetary punishment on green technology innovation effect, but the effect of punishment on institutions is obviously greater than that of punishment on individuals. And the performance of ecological provinces and provinces with better legal environment is also relatively better. Environmental punishment enhances the quantity and quality of green technology innovation through pressure, and improves the quality of green technology innovation through deterrence. Besides, in China, deterrence promotes regional green technology innovation together with the Central Government’s environmental protection inspection, the national green manufacturing strategies and other policies concerned.

## 1. Introduction

At present, global environmental problems are becoming increasingly acute, and carbon peaking and carbon neutrality have become a worldwide hot topic. Green technology innovation is one of the core themes of World City Day just held in Shanghai in 2022. And in the 14th Five-Year Plan and the 2035 Vision Outlines, China swears to build a market-oriented green technology innovation system. In the western countries, market-oriented environment regulation is playing the major rule in the environmental governance. While in China which is characterized of hierarchy bureaucratic governance, environmental punishment is an important means of environmental regulation, which is conducive to regional green transformation and enterprise green technology innovation [1]. China revised Measures for Environmental Administrative Penalties twice in 2010 and 2022. In 2015, Measures for Daily Continuous Penalties Implementation is issued by the environmental protection authorities, which was revised in 2017 (but no revised version has been released so far). According to the manual statistics of the administrative penalties disclosed by the national environmental protection department, since 2010, China has disclosed as many as 520000 environmental administrative penalties. So, how does such frequent environmental punishment affect regional green technology innovation? What is the mechanism behind it?

In the previous research, scholars mainly focus on the relationship between environmental regulation and green technology innovation, as well as the impact of environmental punishment. As green technology innovation has the positive externalities of innovation spillovers and pollution emissions have the negative externalities, these dual externalities will lead market failure and inhibit the green innovation of enterprises [2]. Therefore, environmental regulation is indispensable to improve the efficiency of green technology innovation [1]. Relevant empirical studies indicate that reasonable and appropriate environmental regulation will force enterprises to carry out environment-friendly technological innovation, which will significantly promote the innovation of high-tech equipments and green products [3]. This is consistent with the Porter Hypothesis. While some scholars also believe that environmental regulation will increase the production cost of enterprises, reduce the profit of enterprises, and decrease the green technology innovation incentive of enterprises [4]. In fact, different types of environmental regulations may cause differences in enterprises’ green innovation behaviors. For example, command and control environmental regulation usually makes enterprises’ green technology innovation investment increase first and then decrease, while environmental regulation based on market and public participation will promote enterprises to increase green technology innovation investment after a period of time. Therefore, there may be an inverted U-shape relationship between environmental regulation and green technological innovation [5, 6], or a definite U-shape relationship [7, 8]. In addition, green technology innovations also differ in size and quality, on which the effects of environmental regulation differ [9].

Environmental punishment is an essential policy tool of environmental regulation, aiming to reduce environmental pollution, ecological damage and other illegal acts, so as to implement the national environmental policy [10]. However, in practice, environmental punishment is often considered to be a double-edged sword, which is mainly reflected on the micro level. On the one hand, environmental punishment will cause significant economic losses to individuals and enterprises [11], but courts are more lenient towards environmental violations than towards non environmental violations [12]. Environmental law enforcement is vulnerable to interference from local authorities [13]. In addition, environmental punishment still has a large discretion, which makes its actual effect undermined. For example, environmental violations in some areas continue to persist [14]. On the other hand, environmental violations will be punished in terms of reputation [15], especially when enterprises are punished by the environmental authority, which will lead to a decline in the quality of environmental information disclosure and an increase in financing costs [16]. So, environmental punishment not only makes the target company increase environmental investment, but also has a deterrent effect on peer companies [17]. In 2015, China issued the Environmental Protection Law deemed to be the most stringent administrative law in the field of environmental governance in China history. under strict legal sanctions, enterprises’ responsibility for pollution prevention was strengthened [10], and the number of green patents in heavily polluting industries has significantly increased [18].

However, there are limited studies on the relationship between environmental punishment and green technology innovation, especially in China’s context. From the perspective of intuitive logic, when environmental punishment is weak, pollution enterprises will choose “perfunctory attitude to ignore it” [14], which can neither ease their pollution, nor make a breakthrough in green technology innovation. Too strict environmental punishment may also cause enterprises to face greater public pressure, resulting in production stoppage and a significant decline in their financing capacity [19], thereby hindering enterprises’ green technology innovation. Given the uncertainty of the relationship between environmental punishment and green technology innovation, this paper examines the impact of environmental punishment on regional green technology innovation by utilizing the Sys-GMM method with 30 provincial panel data of China. The contribution of this article lies in: (1) taking a lead-in exploration of the connection between environmental penalties and regional green innovation. Existing researches have focused more on the impact of market-oriented environmental regulation policies on green innovation, while there is a lack of discussion on environmental penalties in developing countries that are biased towards administrative measures. Therefore, it is difficult to gain a comprehensive insight into the innovative effects of different types of environmental regulations. This article reveals the theoretical logic of environmental punishment and green technology innovation, confirms that environmental penalties have a quantity and quality enhancing effect on green technology innovation, and supplements relevant research on the governance effects of environmental regulations. (2) Conducting heterogeneity analysis from multiple perspectives to provide diverse evidences for better leveraging the role of environmental administrative law enforcement in promoting green technology innovation. Existing research mainly explores the heterogeneity of green technology innovation from the perspectives of enterprise size and nature, while paying less attention to the heterogeneity of environmental punishment. This article distinguishes between economic and non-economic penalties, institutional penalties, and individual penalties. More valuable conclusions have been found, which helps to deepen the understanding of the connotation and importance of environmental punishment. (3) Revealing the mechanism of environmental penalties on green technology innovation from the perspectives of pressure channels, deterrence channels, and policy coordination. At present, the main channels for environmental regulation to affect technological innovation are industrial structure, government intervention, FDI, etc. However, there is a lack of attention to the three mechanisms of energy consumption structure (pressure channel), environmental investment from peer enterprises (deterrence channel), and policy synergy (exogenous policy impact). This is also an important finding of this article.

## 2. Theoretical analysis and research hypothesis

### 2.1 Environmental punishment influences regional green technological innovation

Environmental punishment is not only a regulatory tool to reduce environmental law violations and environmental pollution, but also an environmental regulatory means with the functions of advice, guidance, persuasion, etc. Punishment is not the purpose but to urge rectification as soon as possible [15]. On the one hand, environmental punishment not only punishes environmental law violations, but also curbs regional pollutant emission. And it also reduces ecosystem damage and maintains ecosystems’ functions from the perspective of warning, and guides the whole society to attach importance to green development and environmental technology innovation [10]. At the same time, environmental punishment focuses on rectification, which forces enterprises to carry out green technology innovation. When rigidity of environmental regulation increases, enterprises will increase the intensity of green technology innovation investment, thus promoting green technology innovation of enterprises. On the other hand, according to the signal transmission theory and impression theory, the signal of environmental protection penalties will encourage voluntary information disclosure by enterprises [20], and at the same time have a negative impact on corporate financing behavior and capital market performance. Capital providers and investors will make environmental risk assessment and financing decisions according to the severity of environmental penalties imposed on enterprises, which will urge enterprises to pay attention to their own green image and carry out a series of “pro-environment” and “green label” production behaviors. Moreover, according to Porter Hypothesis which is widely used to explain the relationship between environmental regulation and technology innovation, environmental punishment is a regulatory tool of command and control, which urges polluting enterprises to carry out green technology innovation that will offset the costs arising from environmental punishment. Based on this, this paper proposes the following research proposition.

**Hypothesis 1 (H1):** Environmental punishment promotes regional green technology innovation.

### 2.2 Then how does environmental punishment promotes regional green technology innovation?

On the one hand, environmental punishment promotes regional green technology innovation through pressure (urging provincial governments to increase environmental protection expenditure and optimize regional energy consumption structure) and deterrence (prompting peer enterprises to increase environmental protection investment). First of all, environmental punishment also reflects the social pressure faced by regional governments’ environmental governance. Under the present responsibility system of environmental protection objectives since 17^th^ CPC National Congress, provincial governments have to pay attention to green technology innovation, increase environmental protection expenditure, and promote the construction of provincial green technology innovation system. Moreover, environmental punishment will more directly affect the input and output of enterprises, internalize the external costs of polluting enterprises [21], urge enterprises to increase environmental investment, green innovation expenditure and socially responsible investment. Empirical evidence shows that the governance cost of environmental punishment is significantly positively correlated with the R&D investment of regional enterprises [22]. Secondly, environmental punishment increases the consumers’ cost of fossil energy, making economic entities increase the proportion of new energy investment [23], indirectly promoting renewable energy investment and clean energy technology development. At the same time, in China most of the enterprises subject to environmental punishment belong to steel and chemical industries, which are forced to adjust their energy consumption mix, implement clean, low-carbon and intensive energy utilization, and carry out research and development, and develop new and green products. So they can play the main role of green technology innovation. Finally, the “prompt function” of environmental punishment information sends a deterrent signal to the social network, and consequently the perception of those potential violators, namely peer enterprises, on the risk and cost of environmental violations will continue to rise [17]. In order to avoid being punished, peer enterprises inevitably increase environmental protection investment to meet environmental regulation requirements, which actually drive the progress of green technology in the industry. Based on this, this paper proposes the following research propositions:

**Hypothesis 2 (H2):** Environmental punishment promotes regional green technology innovation through pressure and deterrence.

On the other hand, how environmental punishment advances regional green technology innovation is through the synergy of policies concerned. The present political incentives and financial constraints often result in that the local governments are powerless and have no momentum in some aspects of environmental regulation. They weigh the enterprise’s roles in pollution discharge and tax revenue according to the weight of the performance assessment by their superiors or the central governments, which contributes to the discretion of environmental regulation and punishment fluctuates. There is a huge gap between the reality and expectation of environmental policy implementation [14], which leads to biased “environmental punishment”. Recently, China Central Government’s environmental protection inspection is an innovation of the environmental governance model under China’s New Normal Development. This campaign-style governance aims to achieve a dynamic balance between the central authority and local authority, so as to consolidate the authority of the Central Government’s environmental policy [24]. It is hierarchical, binding, and widely participated by the public. Provincial governments dare not bear to tolerate environmental violations, and subsequently environmental punishments are more severe. This combination of Central Government’s campaign-style governance and provincial governments’ normal environmental law enforcement can effectively promote enterprises to shift from speculative environmental protection investment to a green innovation-oriented mechanism, thus improving regional green technology innovation [25]. At the same time, building a green manufacturing system is a strategic task proposed by Made in China 2025, whose subsequent series of incentives not only provide enterprises with a fair competitive environment and institutional guarantee for green manufacturing, but also promote the construction of green factories, green products, green parks and green supply chains, all of which will lead to green technology innovation activities nationwide. In fact, the Implementation Guidelines for Green Manufacturing Engineering clearly put forward to strengthen constraints and strictly implement environmental laws. Then it can be seen that environmental governmental law enforcement and green manufacturing policies will jointly promote regional green technology innovation. Based on this, this paper proposes the following proposition.

**Hypothesis 3 (H3):** The synergy of environmental punishment and policies concerned promotes regional green technology innovation.

**Fig 1:**
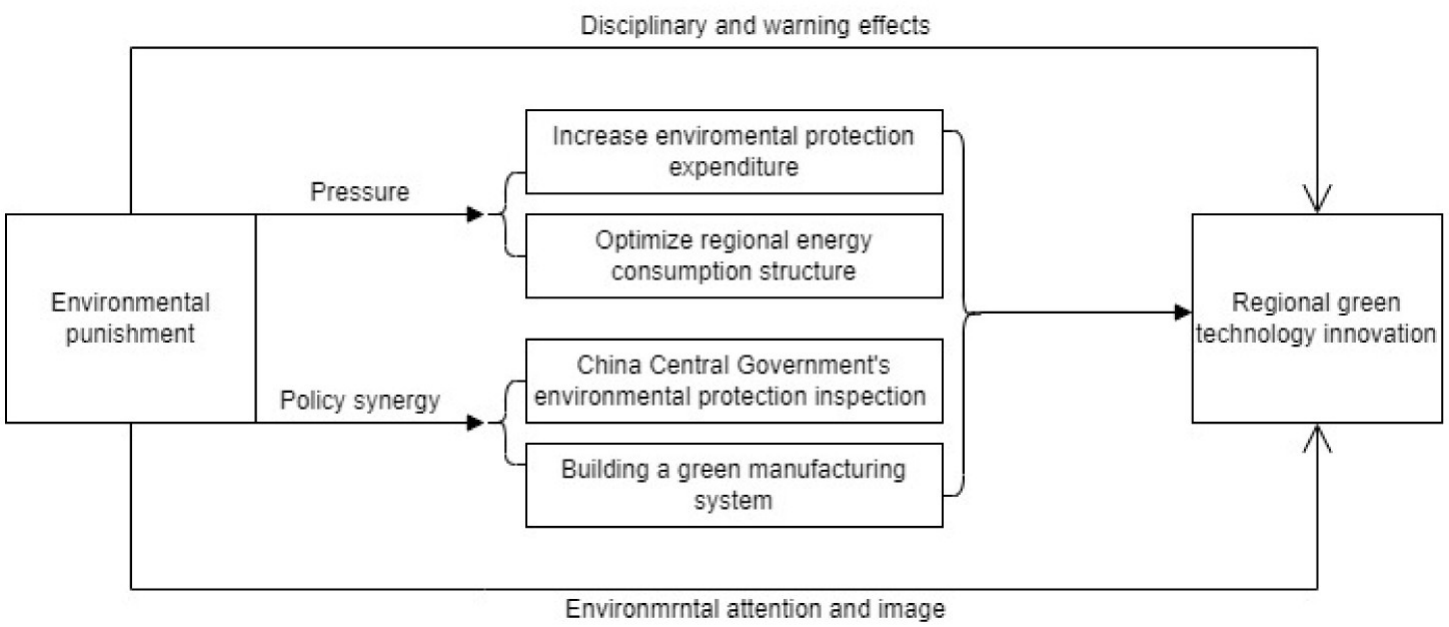
Theoretical framework diagram.

Based the above analysis, we provide the following theoretical framework diagram as shown in Fig 1.

## 3. Empirical strategy: Methods, data and variables

### 3.1 Models

Considering the continuous dynamics of green technology innovation, there may be a reverse causal relationship between green technology innovation and environmental punishment, so the lag term of the explained variable is added to establish a dynamic formula:

$$RGTI_{it}=\mu_{0}+\;RGTI_{it-1}+\alpha_{1}Punish_{it}+\beta Control_{it}+\varepsilon_{it}$$ (1)

Where *i* and *t* indicate the region and year respectively; *RGTI*_*it*_ represents regional green technology innovation; *RGTI*_*it-1*_ is the lagging term of regional green technology innovation; *Punish*_*it*_ represents environmental punishment; *Control*_*it*_ represents control variables, which will be discussed in detail later; *α*_*1*_, *β* refer to the coefficients to be estimated; *μ*_*0*_ is the intercept item; *ε*_*it*_ is the random error term.

In order to further verify H2 and H3, the interaction items are added on the basis of Formula (1) to investigate the mechanism of environmental punishment’s influence on green technology innovation:

$$RGTI_{it}=\mu_{0}+\;RGTI_{it-1}+\alpha_{1}Punish_{it}+\alpha_{2}Crossterm_{it}\times Punish_{it}+\alpha_{3}Crossterm_{it}+\beta Control_{it}+\varepsilon_{it}$$ (2)

Among them, *Crossterm* includes provincial governments’ environmental protection expenditure (PEPE), energy consumption structure (ECS), peer enterprise environmental protection input (PPI), and exogenous policy variable (Post). This paper mainly concentrates on the regression results of the interaction terms, and if the interaction term passes the significance test, it indicates that the environmental protection penalty affects the regional green technology innovation through this factor.

### 3.2 Variables

#### 3.2.1 The explained variable

According to Fang et al. [18], this paper uses Ln (the number of green technology patent applications+1) and Ln (the number of green technology invention patent applications+1) to measure regional green technology innovation scale (RGTI1) and green technology innovation quality (RGTI2) respectively.

#### 3.2.2 Core explanatory variables

Most of the previous literature use dummy variables to measure environmental punishment, and the shortcomings are obvious. The decision of an administrative penalty disclosed by the environmental protection departments has strong authority, and its content includes illegal acts and their evidence, the basis of punishment and the result of punishment. Considering that fines are only one of the types of penalties, and the total number of penalties can cover all types of penalties, we choose the total number of environmental penalties (Punish) to be the proxy variable which is Ln (the total number of environmental penalties in each province+1).

#### 3.2.3 Intermediate variables

Provincial governments’ environmental protection expenditure (PEPE) is represented by “provincial governments’ environmental protection expenditure/the provincial governments’ general fiscal budget expenditure”; energy consumption structure (ECS) by “coal consumption/total energy consumption”, peer enterprises’ environmental protection input (PPI) by “Ln (the number of environmental protection input of listed companies not subject to environmental penalties in each province +1)”, exogenous policy (Post) is a dummy variable, the definition of which is shown in Table 1.

**Table 1:** Variables.

Variable Type	Variable name	abbreviation	definition
The Explained Variables	Scale of green technology innovation	RGTI1	Ln (number of yearly green technology patent applications+1)
	Quality of green technology innovation	RGTI2	Ln (Number of yearly green invention patent applications +1)
Core explanatory variables	Environmental punishment	Punish	Ln (number of environmental punishment in each province+1)
Intermediary variables	Provincial governments’ environmental protection expenditure	PEPE	Provincial governments’ environmental protection expenditure/general budget expenditure of provincial governments
	Energy consumption structure	ECS	Coal consumption/total energy consumption in each province
	Environmental protection investment of peer enterprises	PPI	Ln (Sum of environmental protection investment of listed companies not subject to environmental punishment in each province+1)
Intermediate variables	Central governments’ environmental protection inspector	EI	The year when the central governments’ environmental protection inspectors are stationed is set as 1, otherwise it is 0
	National green manufacturing policy	GM	The province which has issued supporting policies is set to be 1, otherwise 0
Control variables	Regional economic development level	REDL	Ln (Per capita GDP of each province)
	Provincial governments’ innovation support	PGIS	Provincial governments’ science and technology expenditure/general budget expenditure of provincial governments
	Regional R&D	R&D	R&D expenditure/GDP
	Foreign direct investment	FDI	Ln (foreign direct investment)

#### 3.2.4 Control variables

According to the previous research, we select the key factors that may affect regional green technology innovation, namely: regional economic development level (REDL), measured by Ln (per capita GDP of each province); provincial governments’ innovation support (PGIS), measured by the proportion of provincial governments’ science and technology expenditure to provincial governments’ general budget expenditure; regional R&D (R&D), measured by the proportion of R&D expenditure to GDP of each province; foreign direct investment (FDI), measured by Ln (foreign direct investment of each province).

### 3.3 Data source and descriptive statistics

In 2010, the former Ministry of Environmental Protection issued the newly revised Measures for Environmental Administrative Punishment, afterwards the local administrative enforcement of environmental law began to become standardized. So, the starting year of this study is set at 2010. And since the relevant data of each province in 2021 are not issued, the time span is set from 2010 to 2020, and the samples are from China’s 30 provincial jurisdictions except Tibet, Hong Kong, Macao, Taiwan. In terms of data sources, according to the list of green technologies issued by WIPO, the total number of green technology patent applications was identified from the State Intellectual Property Office and attributed to each province. The total number of environmental penalties is manually obtained according to the environmental administrative punishment decision disclosed on the official websites of the provincial environmental protection authorities. The data of other variables are from China Statistical Yearbook, China Energy Statistical Yearbook and CEIC China Economic Database. In this paper, the continuous variables are shrunk by about 1% to remove the influence of extreme values on the conclusions.

Descriptive statistics for each variable are shown in Table 2. As can be seen, the mean values of the scale and quality of green technology innovation are 7.916 and 7.262 respectively, the former is greater than the latter. The standard deviation is 1.366 and 1.410 respectively, the variation coefficient of the former is smaller than that of the latter, which indicates that there is regional heterogeneity of green technology innovation and that the quality of green technology innovation among provinces is greater than the scale of green technology innovation. The average of environmental penalties is 3.999, and the average of environmental penalties in each province in the 11 years after the standardization is 53.5, which shows the high pressure situation of “high frequency and wide coverage” of environmental protection inspection. At the same time, the range between the maximum value and the minimum value is large, and the standard deviation reaches 3.116, indicating that environmental punishment varies greatly among provinces.

**Table 2:** Descriptive statistics.

Variable	Sample	Mean value	Standard deviation	Minimum value	Maximum
RGTI1	330	7.916	1.366	4.477	10.767
RGTI2	330	7.262	1.410	3.807	10.133
Punish	330	3.999	3.116	0	9.280
PEPE	330	3.014	0.938	1.319	5.766
ECS	330	93.92	43.86	6.920	219.3
PPI	330	6.920	4.923	0	17.89
EI	330	0.179	0.384	0	1
GM	330	0.455	0.499	0	1
REDL	330	1.530	0.460	0.519	2.729
PGIS	330	2.070	1.456	0.549	6.248
RD	330	1.081	0.595	0.203	3.017
FDI	330	5.438	1.672	0.565	7.579

## 4. Empirical estimation

### 4.1 Benchmark regression

We use Sys-GMM for regression, and the results are presented in Table 3. From the autocorrelation AR (2) of the disturbance term of each model, its *p* value is big, which means that there is no second-order sequence autocorrelation. At the same time, the *p* value of the Sargan test is bigger than 0.1, which indicates that all tool variables are exogenous, so Sys-GMM estimation is suitable. Lag terms L.RGTI1 and L.RGTI2 is basically significantly positive, indicating that the scale and quality of regional green technology innovation in the previous periods have a positive impact on its present periods, and that green technology innovation is dynamic and cumulative.

**Table 3:** Benchmark regression results.

Variable	(1)	(2)	(3)	(4)
L.RGTI1	0.050** (2.57)		0.022 (0.89)	
L.RGTI2		0.512*** (30.04)		0.520*** (29.91)
Punish	0.334*** (16.00)	0.156*** (18.58)	0.380*** (9.19)	0.129*** (5.53)
Punish^2^			-0.054 (-1.36)	0.033 (1.33)
REDL	0.067*** (2.65)	0.024 (1.02)	0.094*** (4.68)	0.004 (0.20)
PGIS	-0.057 (-1.41)	0.023 (0.92)	-0.010 (-0.25)	0.021 (0.85)
RD	0.410*** (8.45)	0.277*** (16.49)	0.347*** (7.58)	0.278*** (13.52)
FDI	0.182*** (4.66)	0.212*** (8.08)	0.195*** (5.06)	0.206*** (6.37)
_cons	0.010 (0.37)	0.091*** (4.49)	-0.004 (-0.13)	0.068*** (3.12)
P-AR(2)	0.1785	0.6866	0.0951	0.7973
P-Sargan	0.9978	0.9981	0.9988	0.9987
N	300	300	300	300

Note: the standardized regression coefficient is shown in the table;

* * *** And * represent 1%, 5% and 10% probability levels, respectively; the value in parentheses is z, the same below.

Because different indicator dimensions have an impact on the regression coefficient, and for comparison of the regression coefficients of environmental punishment, this paper has standardized all the variables (the same below). It can be seen from Column (1) that the standardized coefficient of Punish is 0.334, and the 1% significance test shows that environmental punishment can increase the scale of regional green technology innovation. At the same time, the regression coefficient of Punish in Column (2) is 0.156, which also has passed the significance test, indicating that environmental punishment can also improve the quality of regional green technology innovation. It can be seen that environmental punishment has the quantity enhancing effect on regional green technology innovation. So H1 proves to be true. Comparing the coefficients of the two, it is self-evident that the quantity enhancing effect of environmental punishment is stronger than the quality enhancing effect. The reason may be that the disciplinary effect of environmental punishment mainly has resulted in the strategic short-term accumulation of green technology innovation, which brings more growth in innovation scale. However, the innovation quality of green technology is difficult and need a long time, and the endogenous drive stimulated by environmental punishment is relatively limited, so its effect on the quality of green technology innovation is relatively weak in present China. As pointed out by Qing et al. [26] and Zhang et al. [27], proactive green innovation is crucial and consideration should be given to how to leverage the long-term effects of environmental punishment.

The conclusion that environmental punishment promotes regional green technology innovation through "incremental improvement" not only reasonably explains the frequent environmental punishment in China in recent years, but also provides empirical evidence for developing countries to implement administrative environmental regulations. This has an important reference value for actively playing the role of government environmental supervision, improving the efficiency of environmental law enforcement, and promoting the reform of the government’s approach to green technology innovation management.

As reported in Columns (3)- (4), the coefficients of the square term have not passed the significance test, while the regression coefficients of the primary item are still significant at the level of 1%, indicating that there is no inverted U-shape nonlinear relationship between environmental punishment and regional green technology innovation at present.

### 4.2 Robustness check

The first way to check robustness is to replace the estimation method. The dynamic panel bias correction LSDV method also has the advantages of Sys-GMM, allowing the lag term of the explanatory variables to be added. From the regression results of Columns (1)-(2) in Table 4, Punish’s standardized regression coefficients were 0.214 and 0.136 respectively, which have also passed the significance test. And the quantity enhancing effect of environmental punishment was still greater than the quality enhancing effect. This is basically consistent with the above conclusions, which shows that the benchmark regression is robust.

**Table 4:** Robustness check.

variable	Bias correction		Replace the explained variable		Excluding the sample in 2020	
	(1)	(2)	(3)	(4)	(5)	(6)
L.RGTI1	0.179*** (2.65)				1.587*** (13.88)	
L.RGTI2		0.414*** (6.11)				1.204*** (20.93)
L. RGTI3			0.766*** (53.58)			
L. RGTI4				0.493*** (13.66)		
Punish	0.214*** (5.77)	0.136*** (4.86)	0.018*** (3.90)	0.014*** (4.53)	0.214*** (9.29)	0.110*** (9.50)
control variable	YES	YES	YES	YES	YES	YES
P-AR(2)			0.6235	0.1502	0.1259	0.9661
P-Sargan			0.9964	0.9968	0.9478	0.9600
N	300	300	300	300	270	270

The second way is to replace the explained variable. In this paper, the total number of green patents granted (RGTI3) and the number of green invention patents granted (RGTI4) in each province in that year are used to replace the number of patent applications, so as to stand for the scale and quality of regional green technology innovation. According to the estimation results in Columns (3)-(4) of Table 4, the positive influence of environmental punishment on regional green technology innovation is similar to that in the benchmark regression, which shows that the above estimations are highly reliable.

The third way is to adjust the time span. Observations of various indicators show that after the outbreak of the COVID-19 in 2020, the number of environmental protection penalties and green patent application in each province declined sharply in comparison with the previous years. In order to eliminate possible errors caused by data fluctuations, this paper excludes the data in 2020 and has conducted regression again. The estimated results are in Columns (5)-(6) of Table 4. It is obvious that environmental punishment’s effect on the green technology innovation is still roughly the same, which proves again that the benchmark regression is robust.

### 4.3 Endogeneity testing

We mainly deal with the endogeneity problems from the following three aspects: firstly, in the benchmark regression, we use the Generalized Method of Moments (Sys-GMM), which not only effectively controls unobservable individual fixed effects through difference, but also uses the lag term of the explanatory variable as the instrumental variable to overcome model endogeneity. From Table 3 above, it can be seen that instrumental variables are effective and have initially alleviated endogeneity issues. Secondly, the propensity score matching method (PSM) is used to control potential sample self-selection problems and further alleviate potential endogeneity impacts. Take the sample value greater than the median of environmental penalty as 1 (treatment group), otherwise as 0 (control group), and perform proximity matching (n = 4), caliper matching, and close-range matching. After the balance testing and system GMM regression based on matching (as shown in Table 5), it was found that the estimated coefficient of environmental punishment is significantly positive at the 1% level, consistent with the previous conclusion. Finally, this article refers to Hering & Poncet’s (2014) [28] research and selects the logarithm of air change rate (lnVent) as the instrumental variable for environmental punishment. The mechanism is that when the air circulation speed is high, pollutants in the area will be quickly pushed away by the wind, reducing the likelihood of environmental punishment due to reduced air pollution. At the same time, it only depends on large-scale climate conditions and weather systems, meeting exogenous conditions. From column (4) of Table 5, it can be seen that in the first stage of regression results, the Vent coefficient is significantly negative, confirming the correlation of instrumental variables. The Cragg Donald Wald F statistics and Sargan statistics indicate that there are no weak instrumental variables or over recognition. Moreover, the regression results of the second stage were consistent with the benchmark results.

**Table 5:** Endogeneity testing.

Variable	PSM			IV_2SLS	
	Proximity Matching = 4	caliper matching	one-to-one ontology match	Punish	GCX1
	(1)	(2)	(3)	(4)	(5)
L.GCX1	0.143*** (3.77)	0.116** (2.09)	0.130** (2.49)		0.247*** (5.93)
Punish	0.121*** (12.34)	0.141*** (17.44)	0.254*** (13.24)		
LnVent				-0.201*** (-3.59)	-0.145*** (-2.76)
control variable	YES	YES	YES	YES	YES
P-AR(2)	0.6923	0.1998	0.8408		0.8356
P-Sargan	0.9999	0.9995	0.9965	0.3910	0.9989
LM				110.89***	
N	160	151	139	330	300

NOTE: Due to the similarity between the estimated results of GCX2 and GCX1, the estimated results of GCX2 is not reported here.

## 5. Further study

### 5.1 Heterogeneity analysis

It has been verified that environmental punishment can improve the scale and quality of regional green technology innovation. So, how to make environmental punishment of Chinese governments and their inspection more reasonable or under what circumstances can environmental punishment better play its part in promoting green technology innovation? The following heterogeneity analysis will provide more evidence and explanation.

#### 5.1.1 Heterogeneity of punishment types

Measures for Environmental Administrative Punishment lists eight types of environmental penalties, including fines, warnings, orders for rectification, etc. Here they are classified into monetary and non monetary penalties. From Columns (1)-(3) in Table 6, it can be seen that the quantity enhancing effect of monetary penalties on regional green technology innovation is equivalent to that of non monetary penalties. The comparison between Columns (2)-(4) also shows that the quality enhancing effect of monetary penalties on green technology innovation is not much different from that of non monetary penalties. For a period of time in China, environmental authorities were keen on “punishment instead of management” and fell into a vicious circle of “pollution—fine—repollution—refine”. However, our estimations show that non monetary punishment also has a strong effect (even slightly stronger than monetary punishment), so these two types of punishment should be combined. In fact, in recent years, environmental punishment has paid more and more attention to process rectification since a single file cannot produce the expected corrective effect. As the Measures for Ecological Environment Administrative Punishment (Draft waiting for review) in 2022 shows, those who violate it for the first time and without subjective faults may not be punished, but instead be urged to rectify as soon as possible. This may better facilitate regional green technology innovation.

**Table 6:** Heterogeneity of types and objects of environmental punishment.

Variable	Monetary punishment		Non monetary punishment		Punishment upon institutions		Punishment upon individuals	
	(1)	(2)	(3)	(4)	(5)	(6)	(7)	(8)
L.RGTI1	0.137*** (8.11)		0.085*** (3.29)		0.047** (2.46)		0.323*** (20.93)	
L.RGTI2		0.565*** (31.54)		0.516*** (31.59)		0.511*** (30.36)		0.706*** (52.33)
Punish	0.282*** (17.76)	0.144*** (18.16)	0.287*** (12.80)	0.146*** (15.86)	0.337*** (15.98)	0.158*** (18.70)	0.051** (2.42)	0.002 (0.15)
control variable	YES	YES	YES	YES	YES	YES	YES	YES
P-AR(2)	0.1163	0.7019	0.4088	0.9061	0.1967	0.7112	0.0073	0.3765
P-Sargan	0.9976	0.9983	0.9978	0.9981	0.9977	0.9981	0.9975	0.9975
N	300	300	300	300	300	300	300	300

#### 5.1.2 Heterogeneity of punishment objects

The parties involved in environmental violations can be individuals, legal persons and social organizations, then the punishment can be divided into punishment upon institutions and punishment upon individuals. At present, only enterprises are punished in China, and the punishment upon individuals are often only aimed at the citizens’ environmental violation of laws. From Columns (5)-(6) of Table 6 and Columns (7)-(8) of Table 6, it can be found that the quantity enhancing effect induced by punishment upon institutions is significantly greater than punishment upon individuals. This is because legal persons and social organizations are the main bodies of regional green technology innovation, and also the key objects of environmental punishment in present China. When they are punished for environmental violation, their production and operation and social reputation will be affected, and economic losses and social capital losses will occur. The results here indicate that governments should continue to strengthen the punishment upon institutions who violate the environmental laws or regulations, consolidate the “strict inspection and heavy punishment” for responsible persons of enterprises, and implement the “double punishment system” as soon as possible, so as to better play their regulatory roles and facilitate regional green technology innovation.

#### 5.1.3 Heterogeneity of ecological provinces

Considering that environmental regulation and green technology innovation are closely related to the strength and breadth of provincial governments’ ecological construction, it is necessary to test those provinces that have implemented the ecological civilization construction in their entire jurisdiction. The ecological province construction strategy was initiated in 1999 in China, which has lasted for a long time and covered the most provinces. With 16 provinces approved as piloting ones, this strategy has achieved outstanding results and driven China’s overall green transformation. Therefore, we separate the research samples into ecological provinces and non ecological provinces to explore the heterogeneous impact of environmental punishment on green technology innovation. From Columns (1)-(4) in Table 7, it can be seen that the coefficients of environmental punishment in ecological provinces is greater than those in non ecological provinces. That is, the environmental punishment in ecological provinces can produce stronger quantity enhancing effect of green technology innovation, and correspondingly the quality enhancing effect of ecological provinces is better. The reason is that the ecological provinces can make full use of the advantages of the province-wide reform, create a good social atmosphere and regulatory environment, mobilize the enthusiasm of all partners, and amplify the green technology innovation effect of environmental punishment, thus improving their overall green technology innovation.

**Table 7:** Heterogeneity between the (non)-ecological provinces and heterogeneity of marketization.

Variable	Ecological province		Non ecological province		Province with good law environment		Province with poor law environment	
	(1)	(2)	(3)	(4)	(5)	(6)	(7)	(8)
L.RGTI1	0.162** (2.19)		0.213*** (3.37)		0.053*** (2.70)		0.048*** (2.20)	
L.RGTI2		0.476*** (11.84)		0.101** (2.08)		0.290*** (9.32)		0.364*** (3.38)
Punish	0.367*** (5.09)	0.217*** (6.60)	0.214*** (6.75)	0.045 (1.47)	0.590*** (9.51)	0.388*** (7.30)	0.443*** (10.03)	0.160*** (5.51)
control variable	YES	YES	YES	YES	YES	YES	YES	YES
P-AR(2)	0.1636	0.2881	0.5021	0.1084	0.2491	0.4326	0.9872	0.5502
P-Sargan	1.0000	1.0000	1.0000	1.0000	1.0000	1.0000	1.0000	1.0000
N	160	160	140	140	150	150	150	150

#### 5.1.4 Heterogeneity of provinces with different market law environments

Environmental punishment is not an absolutely independent factor, and the effectiveness of environmental law enforcement is highly dependent on the provincial legal environment. Moreover, the legal environment is crucial to the protection of green intellectual property rights, and also a pillar for building a market-oriented green technology innovation system. So this paper measures regional legal environment based on the Development Scores of Market Intermediary Organizations and the Legal System in the Report on China’s Provincial Marketization Index, and groups them according to the geometric average of each province. The top 50% are the groups with good legal environment, and the bottom 50% are the groups with poor legal environment. As can be seen from the Punish’s standardized coefficients of Columns (5)-(8) in Table 6, the quantity enhancing effect generated by environmental punishment in provinces with good legal environment is better than that of provinces with poor legal environment, and the quality enhancing effect of the former is 2.4 times that of the latter. It can be seen that accelerating the construction of laws concerned is also important to enhance the role of environmental regulation in regional green technology innovation.

### 5.2 Mechanism of intermediate effect

In order to test H2, we will further test the mechanism of intermediate effect of environmental punishment affecting regional green technology innovation. It can be seen from Columns (1)-(2) in Table 8 that the standardized coefficients of the crossterm PEPE×Punish are 0.126 and 0.071 respectively. Only the former has passed the significance test, indicating that environmental punishment can have the quantity enhancing effect of regional green technology innovation by increasing provincial environmental protection expenditure, but can not produce significantly the quality enhancing effect. It can be observed that the pressure mechanism of environmental protection expenditure induced by environmental punishment is partially true. The pressure is transmitted to provincial governments through environmental law enforcement, forcing them to increase environmental governance expenditure, thus improving the scale of regional green technology innovation. However, this pressure failed to effectively drive economic entities to carry out green high-technology research and development. Therefore, relying on a single environmental punishment method to promote regional green technology innovation not only poses obvious bottlenecks, but also makes it difficult to form a sustainable long-term mechanism.

**Table 8:** Mechanism of intermediate effect.

Variable	Pressure channel: environmental protection expenditure		Pressure channel: energy consumption structure		Deterrence channel: peers’ investment in environmental protection	
	(1)	(2)	(3)	(4)	(5)	(6)
L.RGTI1	0.123*** (4.23)		0.050*** (3.74)		0.114*** (3.53)	
L.RGTI2		0.469*** (10.04)		0.558*** (27.56)		0.428*** (11.93)
Punish	0.347*** (8.92)	0.237*** (5.15)	0.124*** (3.14)	0.085** (2.22)	0.265*** (12.02)	0.130*** (7.55)
PEPE × Punish	0.126** (2.03)	0.071 (1.54)				
PEPE	0.190*** (8.00)	0.106*** (3.16)				
ECS × Punish			0.278*** (5.35)	0.074** (2.01)		
ECS			-0.474*** (-7.74)	-0.330*** (-6.44)		
PPI × Punish					0.011 (0.48)	0.036** (2.12)
PPI					0.055*** (3.35)	0.044** (2.29)
control variable	YES	YES	YES	YES	YES	YES
P-AR(2)	0.6129	0.5119	0.1698	0.2874	0.0839	0.4520
P-Sargan	0.8980	0.8978	0.9072	0.9003	0.9988	0.8385
N	300	300	300	300	300	300

As Columns (3)-(4) have reported the mechanism of intermediate effect of the variable ECS, environmental punishment has produced the quantity enhancing effect on regional green technological innovation by pushing the adjustment of the energy consumption structure, and the quantity enhancing effect is greater than the quality enhancing effect. It is shown that the pressure mechanism of energy consumption structure adjustment caused by environmental punishment is true. It encourages and forces illegal enterprises’ use of cleaner and sustainable energy, thus promoting the scale and quality of regional green technology innovation. Estimates (5) and (6) show that environmental punishment can deter peer enterprises and urge them to increase investment in environmental protection and actively establish independent green technology systems, thus significantly improving the quality of regional green technology innovation (rather than the scale). This means that the peer deterrent effect of environmental punishment has a unique value for the quality of green technology innovation in a region by setting an example to others. In summary, H2 is generally true.

### 5.3 The mechanism of *Punish*’s synergy with policies concerned

In recent years, more and more scholars have incorporated exogenous policies into empirical models to assess the possible synergistic effects of policies. Therefore, according to H3, this paper also includes the policy variable Post to check the mechanism. In China, the Central Governments’ Environmental Protection Inspection was launched as a pilot project in Hebei in January 2016. Later in June, the first batch of inspectors to eight provinces was sent out. By March 2022, two rounds of 10 batches of inspectors had been stationed nationwide. Here the paper defines Post as 1 if there are inspectors stationed in that province during this period. Otherwise it is set as 0. The crossterm EI × Punish represents the combination of Central Governments’ campaign governance such as Environmental Protection Inspection and provincial governments’ conventional environmental law enforcement, which is assumed to have an impact on regional green technology innovation. As can be seen from the Columns (1)-(2) in Table 9, the standardized coefficients of the crossterm are significantly positive, indicating that the overall binding force jointly caused by the Central Governments’ environmental protection inspection and provincial governments’ environmental administrative law enforcement has promoted the scale and quality of regional green technology innovation, and that the quantity enhancing effect is greater than the quality enhancing effect. So, Central Governments’ environmental protection inspection should be embedded in the overall plan of provincial green technology innovation.

**Table 9:** Mechanism of Punish’s synergy with policies concerned.

Variable	Central Governments’ environmental protection inspection		National green manufacturing policy	
	(1)	(2)	(3)	(4)
L.RGTI1	0.073*** (2.65)		0.032* (1.80)	
L.RGTI2		0.448*** (13.05)		0.480*** (15.18)
Punish	0.283*** (15.08)	0.142*** (8.92)	0.290*** (15.19)	0.139*** (10.55)
EI × Punish	0.086*** (8.51)	0.027*** (3.03)		
GM × Punish			0.063*** (6.95)	0.066*** (11.74)
control variable	YES	YES	YES	YES
P-AR(2)	0.4713	0.6175	0.2793	0.9810
P-Sargan	0.9988	0.9988	0.9986	0.9987
N	300	300	300	300

Also in China, national strategies such as Made in China 2025 and Industrial Green Development Plan (2016–2020) have sworn to build a green manufacturing system, implement green manufacturing and lend support of special construction funds, green credit and the like. Each province has also introduced correspondent incentive policies since 2016, so the Post in 2016 and afterwards is set as 1, and the Post before 2016 is set as 0. The coefficients of the crossterm GM × Punish represents the synergistic effect of green manufacturing policies and environmental punishment on regional green technology innovation. From Columns (3)-(4), it can be seen that the national green manufacturing policies and the incentives stimulated by environmental administrative law enforcement work together to produce the quantity enhancing effect on regional green technology innovation. This proves that H3 stands. But, this time the quality enhancing effect is slightly greater than the quantity enhancing effect, and the coefficient compared with that of Column (2) has greatly improved, which shows that it is difficult to produce a strong quality enhancing effect although the rigid environmental constraint is important. However, after adding the incentive policy, difference occurs, that is, the quality enhancing effect of green technological innovation has risen.

## 6. Conclusions and suggestions

This paper matches the manual data of administrative penalties from environmental protection authorities with the provincial panel data of China, and uses the Sys-GMM method to test the impact of environmental punishment on regional green technology innovation. The results show that: (1) Environmental punishment can significantly enhance the quantity and quality of regional green technology innovation, and the quantity enhancing effect is greater than the quality enhancing effect, which stands still true after several robustness tests; (2) Further estimates show that monetary punishment and non monetary punishment have the same effect on provincial green technology innovation, but the quantity enhancing effect of green technology innovation induced by punishment on institutions is significantly greater than that of punishment on individuals, and environmental punishment has a strong green technology innovation effect, especially the quality enhancing effect, and especially in ecological provinces and provinces with better legal environments; (3) Environmental punishment urges provincial governments to increase environmental protection expenditure and optimize energy consumption structure through pressure, resulting in its quantity enhancing effect of green technology innovation, and prompt peers to increase environmental protection investment through deterrence, resulting in its quality enhancing effect of green technology innovation; (4) The Central Government’s Environmental Protection Inspection and the national green manufacturing policies, together with environmental administrative law enforcement such as environmental punishment, have jointly promoted regional green technology innovation.

Based on the above analyses and conclusions, this paper has the following suggestions for environmental governance. First, the environmental punishment and regional green technology innovation should be coordinated. For example, in the newly revised Administrative Penalty Measures for Ecology and Environment, the double carbon strategy is highlighted to grant economic entities with the green development responsibilities and obligations. Environmental Protection Inspection by the Central Government and provincial law enforcement should be incorporated into the regional green technology innovation system. Second, it is necessary to enhance the rigid constraint and deterrent effect of environmental punishment. With “urging rectification as soon as possible” at the core, China’s central environmental authorities should strengthen environmental punishment, urge provincial governments to increase environmental protection expenditure, accelerate the development and utilization of clean energy, and improve the pertinence, accuracy and effectiveness of environmental punishment, so as to drive peer enterprises to increase environmental technology R&D investment. Third, the synergic roles of the Central Government’s Environmental Protection Inspection and green manufacturing incentive policies should be fully exercised. The “central inspection, enterprises’ rectification and public participation” should be combined with provincial environmental law enforcement. The Central Government’ environmental protection inspection should be especially aimed at tackling the long-standing local environmental headaches in China’s present bureaucratic command-control governance of the environment. Environmental punishment should be coupled with implementing incentive policies such as green manufacturing ones so as to jointly promote regional green innovation and development.

It should be pointed out that due to the limitations of data availability, this article only explores the impact of environmental penalties on regional green technology innovation at the provincial level. The research scale is relatively large, and the author will make improvements in future research, such as aggregating environmental penalty data of listed companies into urban data, and then conducting research on cities.

## Supporting information

S1 File (XLS)
